# Combating noncommunicable diseases in a digital world: Youth shaping the future through digital citizenship for health

**DOI:** 10.1371/journal.pdig.0000854

**Published:** 2025-04-29

**Authors:** Salman Fitrat Khan, Aditi Jhaveri

**Affiliations:** 1 Grant Government Medical College and Sir J J Group of Hospitals, Mumbai, India; 2 Department of Screening and Early Detection, Karkinos Healthcare Pvt. Ltd., Mumbai, India; McGill University, CANADA

## The growing burden of NCDs in LMICs

Noncommunicable diseases (NCDs) accounted for 75% of global deaths unrelated to pandemics, with nearly 43 million deaths in 2021 [1]. In low- and middle-income countries (LMICs), where 82% of these deaths occur prematurely [1], the rising tide of NCDs is not just a health issue—it’s a generational emergency. With growing digital capacity and youth literacy, countries like India have a unique opportunity to address the rising burden. Digital health (DH) can transform prevention, management, and treatment in LMICs facing limited resources. Central to this change is Digital Citizenship for Health (DC4H) [2], a framework that empowers communities, particularly youth, to shape healthcare responses that are relevant, equitable, and sustainable. NCDs require sustained care, which traditional health systems in LMICs often lack. Youth-co-designed Digital Tools and Interventions (DTIs) can bridge this gap with continuity, personalization, and access.

## Reimagining healthcare governance through DH

DH signifies a major shift in healthcare governance. Historically, decisions on healthcare delivery, technology development, and data management have been made by a few key stakeholders. While DTIs are proliferating, their development often remains exclusionary. Democratizing these decisions is key to building equity in health systems. This means ensuring that all individuals across society, particularly marginalized groups, are empowered to contribute to policy discussions, technology development, and data governance.

## Youth as drivers of NCD-focused digital innovation

Young people bring lived experience and digital fluency. Their engagement in NCD-focused digital innovation is essential to achieving health equity. Through DC4H, youth can advocate for national policies that prioritize NCD prevention, including the integration of digital screening for hypertension and diabetes at the primary care level. In practice, youth can participate in policy forums, advocacy campaigns, and design workshops, where they actively contribute ideas for DH solutions, ensuring that technologies and strategies are inclusive, accessible, and relevant.

Youth can help ensure DH tools are usable, relevant, and culturally appropriate. Supporting youth-led innovations through hackathons, seed funding, and fellowships can improve digital solutions for NCDs, especially in mental health, obesity, and substance use. Youth can address low user engagement by designing tools that foster usability and habit formation.

## Inspirations from India and potential for personalized NCD care

In India, initiatives such as the Ayushman Bharat Digital Mission [3] have set the stage for a digitally driven healthcare experience, yet there remains considerable room for youth engagement [4]. As digital natives, youth are uniquely positioned to lead the creation of innovative, scalable health solutions. Their involvement ensures digital tools are accessible and user-friendly. A standout example is the student-initiated Khushi Baby Project in Rajasthan [5], India, which enables Community Health Workers (CHWs) to use mobile technology and machine learning to prioritize high-risk individuals, improve prenatal care adherence, and enhance family planning access. By leveraging mobile phones, these workers can provide more personalized care, ensuring early diagnoses, effective management, and continuous monitoring. Though not originally for NCDs, Khushi Baby’s model of CHWs using mobile data to flag risk and provide targeted care can be adapted.

Such initiatives show the power of digital tools in low-resource settings. Moreover, youth participation in these projects is vital. Too many NCD solutions are siloed—used only by clinicians or “when things get bad.” But young people use tech daily. We need to embed digital NCD interventions into platforms youth already use to build preventive health habits in everyday life. Additionally, gamified fitness applications or artificial intelligence (AI)-driven nudges for medication adherence can promote long-term engagement in care.

A major challenge in DH is the ethical and transparent use of technologies such as AI and data analytics. As data privacy and AI governance gain importance, youth should lead efforts to ensure responsible use. Youth must be empowered to shape conversations on transparency, privacy, and bias in algorithmic decision-making. For example, digital risk-scoring tools for cardiovascular disease may inadvertently reflect socioeconomic or gender biases unless developed with community input.

Youth can advocate for policies that promote transparency, accountability, and inclusivity in the collection, storage, and use of health data. Youth engagement in AI and data privacy helps ensure DH protects rights while supporting innovation. They can co-develop guidelines to reduce bias in AI and advocate for secure data practices. Youth can also co-develop or test in their communities simple machine-learning models that flag cardiovascular risk or diabetes progression, enhancing community-based early detection. This involvement promotes ethical, equitable, and accountable DH tools.

The future of healthcare lies in the intersection of technology and community governance. DH solutions are not merely about adopting new technologies but keeping communities at the heart of digital transformations. Ensuring that youth is not tokenized and restricted to unfeasible innovative solutions but is rather meaningfully included in the existing policy and governance structures is crucial to achieving this transformation [6].

## Toward a digitally empowered, NCD-responsive health system

The WHO’s Package of Essential NCD Interventions suggests [7] expanding the use of DTIs to increase access to NCD prevention, improve early diagnosis, and reduce costs. This is particularly important in LMICs, where healthcare resources are limited. Initiatives like eSanjeevani [8], India’s telemedicine platform, have demonstrated the potential of DTIs to expand healthcare access, serving over 275 million patients since its launch. The success of mDiabetes [9] demonstrates that low-cost text message interventions (approximately $0.50 per person) can effectively drive population-level behavior change for NCD prevention in LMICs. Similarly, platforms like HealthifyMe [10], Fit India Movement [11], and Quit Smoking apps empower individuals to monitor their health, adhere to treatment plans, and self-manage chronic conditions. However, access without youth engagement is a lost opportunity in combating NCDs. Youth can be empowered as **Digital NCD Fellows**—helping primary health centers use tech more effectively, training peers in self-monitoring, and translating data into action.

## Conclusion

Ultimately, DC4H enables youth to engage in health policy, technology development, and data governance. Their inclusion promotes a more ethical, transparent, and inclusive health system that reflects diverse community needs. Youth engagement in DH must move beyond pilot projects and panels. We need to invest in youth leadership pipelines within public health systems through internships, policy roles, innovation grants, and capacity building. The fight against NCDs can only be won if the generation most affected is also the one designing the response, and for that, we can’t tokenize youth; we must empower them.

## Abbreviations:

AI: artificial intelligence
CHWs: Community Health Workers
DC4H: Digital Citizenship for Health
DH: digital health
DTIs: Digital Tools and Interventions
LMICs: low- and middle-income countries
NCDs: noncommunicable diseases
